# Comprehensive analysis of phenolic profile, their antioxidant activities, and physicochemical characteristics of rapeseed oil under various microwave and storage conditions

**DOI:** 10.1016/j.fochx.2025.102637

**Published:** 2025-06-11

**Authors:** Yao Zhang, Zhixian Xiang, Rong Xia, Wenxi Chen, Xin Zhang, Hongting Lei, Fang Wei, Yongjun Yuan

**Affiliations:** a Chongqing Key Laboratory of Speciality Food Co-Built by Sichuan and Chongqing, School of Food and Bioengineering, Xihua University, Chengdu 610039, China; b Key Laboratory of Grain and Oil Processing and Food Safety of Sichuan Province, College of Food and Bioengineering, Xihua University, Chengdu 610039, China; c Oil Crops Research Institute of Chinese Academy of Agricultural Sciences, Key Laboratory of Oilseeds Processing of Ministry of Agriculture, Hubei Key Laboratory of Lipid Chemistry and Nutrition, Wuhan 430062, China; d Hubei Hongshan Laboratory, Wuhan 430070, China

**Keywords:** Rapeseed oil, Targeted metabolomics, Phenolic profile, Microwave pretreatment, Antioxidant activities

## Abstract

Rapeseed oil (RO) is a widely consumed edible oil in China, valued for its nutritional benefits and oxidative stability. Phenolic compounds in RO function as natural antioxidants, enhancing both its health-promoting properties and shelf life. In this study, a targeted metabolomics method was developed and validated for the quantification of 24 phenolic compounds. The method demonstrated high accuracy, with determination coefficients exceeding 0.99. Chemometric analysis revealed that microwave power exerted a greater impact on phenolic profile of RO than storage duration. Specifically, higher microwave powers and shorter storage periods associated with increased content of most phenolics. Sinapic acid emerged as a key marker distinguishing microwave-pretreated ROs. The most significant pathway associated with phenolics was flavone and flavonol biosynthesis. Changes in antioxidant capacity, color, oxidation indicators, and fatty acid composition of RO following various microwave and storage conditions were anatomized. Strong relationships were observed between phenolic profile and antioxidant activity, color, and oxidation indicators. These findings provide valuable insights into the nutritional evaluation and shelf-life prediction of RO, providing a foundation for optimizing processing and storage conditions to preserve its quality and functionality.

## Highlights

• Phenolic profile of rapeseed oil (RO) was detected by targeted metabolomics.
• Microwave pretreatment had more effects on phenolics than storage.
• Sinapic acid emerged as a key marker distinguishing microwave-pretreated ROs.
• Antioxidant capacity and redness of RO were positively correlated with phenolics.

## 1 Introduction

Rapeseed oil (RO) is one of the most widely consumed edible oils in China and the third most produced vegetable oil in the world, following palm and soybean oils (Zhang et al., 2024). Derived from the seeds of *Brassica* species, such as *Brassica rapa* L. and *Brassica napus* L., RO is traditionally manufactured through rapeseed roasting and pressing, resulting in a nutrient-rich oil with a distinctive flavor (Chew, 2020). It is particularly popular in southern Chinese provinces, such as Sichuan, Hunan, Hubei, Yunnan, and Guizhou. RO is notable for its high content of unsaturated fatty acids, particularly oleic acid (C18:1, 50 %–70 %), linoleic acid (C18:2, 15 %–30 %), and linolenic acid (C18:3, 5 %–14 %). This balanced fatty acid profile, with an optimal ratio of C18:2 to C18:3 (2:1), contributes to its excellent digestibility and absorption rate, which approaches nearly 99 % (Wroniak, Rękas, Siger, & Janowicz, 2016). Additionally, RO contains a variety of bioactive ingredients, such as phenolic compounds, tocopherols, squalene, phytosterols, and carotenoids. Among these, phenolic compounds have aroused increasing attention due to their diverse health-promoting properties, including antioxidant, anti-inflammatory, anticancer, and antimicrobial activities. These compounds are particularly recognized for their ability to enhance plasma antioxidant capacity, mitigate oxidized low-density lipoprotein levels, and potentially reduce risks of cardiovascular and cerebrovascular diseases (Eghlima, Sonboli, & Mirjalili, 2025; Rudrapal et al., 2022; Wang et al., 2021; Wang et al., 2021; Yu, Yu, Ma, & Li, 2021).

Recent studies have demonstrated that microwave pretreatment significantly enhanced both the total phenolic content (TPC) and oxidative stability of RO by breaking down cellular membranes, inducing permanent pore formation in rapeseeds, and promoting the release of bound phenolic compounds into the oil (Özcan & Uslu, 2023). Notably, microwave pretreatment has been found to decarboxylate non-free phenolic compounds, converting them into free phenolic forms. For instance, Cong, Zheng, Huang, Liu, and Zheng (2020) reported that microwave pretreatment increased canolol content while reduced levels of sinapic acid derivatives in RO. Furthermore, the storage stability of oil is determined by its initial antioxidant and fatty acid composition, as well as exposure to oxidative factors such as light and oxygen (Alonso-Salces et al., 2021). Calatayud, Li, Brizzolara, Tonutti, and Wang (2024) observed that higher phenolic content in olive oil was associated with reduced oxidation during storage, with specific phenols serving as reliable indicators of oil quality and shelf life. Similarly, Monasterio et al. (2023) identified hydroxytyrosol as a potential ageing marker for virgin olive oil. Despite these advances, the effects of microwave pretreatment on other phenolic compounds, as well as the dynamic changes in phenolic profiles and physicochemical properties of RO during storage, remain insufficiently explored. Developing a highly sensitive analytical method could provide deeper insights into the variations of phenolic compounds across different RO samples, offering a more comprehensive understanding of how processing and storage conditions influence the oil's nutritional quality and stability.

Liquid chromatography-tandem mass spectrometry (LC-MS/MS)-based targeted metabolomics has emerged as a powerful tool for phenolic compound analysis due to its exceptional selectivity, sensitivity, and robust quantitative capabilities (Cao et al., 2020). For example, Wang, Zhang, Yu, Ma, and Li (2021) used LC-MS/MS to simultaneously quantify 13 phenolic compounds in rapeseed, Miho et al. (2021) applied targeted metabolomics to evaluate phenolic profiles of 44 virgin olive oil cultivars. Similarly, Yu et al. (2021) developed a rapid and accurate analytical method combining mixed-mode solid-phase extraction with chemical labeling-assisted LC-MS for phenolic compounds in RO. These applications highlighted the effectiveness of LC-MS/MS-based targeted metabolomics in achieving precise and quantitative analysis of phenolic compounds.

In this study, a highly selective and accurate LC-MS/MS-based targeted metabolomics method was developed and validated for the simultaneous quantification of 24 phenolic compounds. This method was subsequently applied to analyze the phenolic profiles of RO subjected to various microwave pretreatment conditions and storage durations. Additionally, changes in antioxidant capacity, color, TPC, oxidation indicators, and fatty acid composition of RO were systematically examined. Correlation analysis was employed to reveal the relationships between the phenolic profiles, antioxidant capacity, color, and oxidation indicators of RO, providing a deeper understanding of how processing and storage conditions affect the phenolic profile and overall quality of RO.

## 2 Materials and methods

### 2.1 Materials

The rapeseed used in this study was the Yuyou 35 variety, harvested in May 2024 from Suining, Sichuan Province, China. The seeds exhibited a moisture content of 12.75 ± 0.42 % and fat content of 43.78 ± 1.68 %. For phenolic compound analysis, we employed 24 authentic standards (detailed in **Table S1**, Supporting Information). Stock solutions (1 mg/mL) of each phenolic standard were dissolved in methanol and stored in brown bottles at −20 °C, with working solutions freshly prepared by methanol dilution prior to analysis. All reagents were of analytical grade unless otherwise specified. The Folin-Ciocalteu phenol reagent was purchased from Yuanye Bio-Technology Co., Ltd. (Shanghai, China). Saturated sodium carbonate and n-hexane were obtained from Sinopharm Chemical Reagent Co., Ltd. (Shanghai, China). LC-MS grade solvents included methanol (Fisher Scientific, USA) and acetonitrile (Merck, Germany). Formic acid (HPLC grade) was purchased from ANPEL Laboratory Technologies Inc. (Shanghai, China), and ammonium formate (LC-MS grade) was purchased from Aladdin (Shanghai, China). For antioxidant capacity assays, 2,2-diphenyl-1-picrylhydrazyl (DPPH, 95 %), 2,2′-azino-bis (3-ethylbenzothiazoline-6-sulfonic acid) diammonium salt (ABTS, 95 %), and 6-hydroxy-2,5,7,8-tetramethylchromane-2-carboxylic acid (Trolox, 97 %) were purchased from Sigma-Aldrich (Poznań, Poland). Ultrapure water (18 MΩ·cm^−1^) was obtained using a Milli-Q water purification system (Millipore, USA).

### 2.2 Preparation of RO samples

For microwave pretreatment, 500 g of cleaned rapeseeds were weighed and subjected to a commercial microwave oven (M1-L202B, Midea, China) at 2450 MHz. Five power levels (70, 210, 350, 560, and 700 W) were applied for 12 min each, with untreated rapeseeds serving as controls. To avoid localized overheating, we implemented intermittent stirring every 2 min, along with rapid temperature measurements, repeating this process six times over a total microwave duration of 12 min. And the temperature of rapeseed after different microwave times was shown in **Fig. S1**. After treatment, rapeseeds were quickly cooled to room temperature. All rapeseed samples, including controls, were then cold-pressed at 635 W using an oil expeller (D02, DONGDUBAO, China) to obtain crude RO. The crude oil was clarified by centrifugation (5000 rpm, 10 min) to remove impurities, and the purified oil was transferred into brown glass bottles stored at −20 °C until analysis. To evaluate oxidative stability, Schaal oven test was employed under accelerated conditions. The RO samples were sealed in brown glass bottles and incubated in a constant temperature oven (GZX-9140MBE, BOXUN, China) maintained at 63 ± 0.5 °C. Sampling occurred at 5-day intervals over a 25-day period (0, 5, 10, 15, 20, and 25 days) to monitor progressive changes in oil quality parameters.

### 2.3 Extraction of phenolic compounds

Phenolic compounds were extracted based on the method proposed by Zhang et al. (2023) with modifications. Briefly, 0.5 g of RO was accurately weighed into a centrifuge tube and mixed with 1.0 mL of methanol. The mixture was vortexed (2000 rpm, 30 s) followed by ultrasonic extraction (SB-5200DTN, XINZHI, China) at 4 °C for 2 min. After overnight incubation at −20 °C, samples were centrifuged at 13,000 rpm for 5 min. The supernatant was collected, evaporated to dryness under nitrogen gas, and reconstituted in 100 μL of methanol. The reconstituted solution underwent a final centrifugation step (13,000 rpm, 5 min), after which the clarified supernatant was transferred for further analysis.

### 2.4 Identification and quantification of phenolic compounds

Phenolic compounds in RO were analyzed using a validated LC-MS/MS targeted metabolomics approach. The analytical system comprised a Shimadzu LCMS-8045 triple quadrupole mass spectrometer equipped with an electrospray ionization (ESI) interface and a Shimadzu HPLC-20 CE system. The HPLC system included 20 CE binary pumps, a SIL-20 AC autosampler, a CTO-20 A column oven, and a DGU-20A3R degassing unit. Chromatographic separation was achieved using a Shim-pack GIST-HP C18 column (2.1 mm × 100 mm, 3.0 μm, Shimadzu, Japan) maintained at 40 °C. The mobile phases consisted of water containing 2 mmol/L ammonium formate and 0.02 % (v/v) formic acid (phase A) and acetonitrile (phase B). A 6-min gradient elution program at a flow rate of 0.3 mL/min was employed for the separation of phenolic compounds. The gradient for phase B was as follows: initiated at 30 % (0–0.2 min), increased linearly to 80 % (0.2–4.0 min), held for 1 min (4.0–5.0 min), then returned to initial conditions (5.0–5.2 min) followed by 0.8 min re-equilibration. The inject volume was 2 μL. Mass spectrometric parameters were optimized for the detection of phenolic compounds, with interface voltage set to 3.0 kV, interface temperature at 300 °C, nebulizer gas flow rate of 3 L/min, drying gas flow rate of 10 L/min, and heating gas flow rate of 10 L/min. Collision energy (CE) and Q_1_/Q_3_ pre-filters were optimized via direct infusion mode of LC-MS. Phenolic compounds were analyzed using multiple reaction monitoring (MRM) mode, with MRM transitions for the 24 phenolic compounds detailed in **Table S2** (Supporting Information).

The method was rigorously validated for its performance in terms of linearity, limits of detection (LODs), limits of quantitation (LOQs), and precision (intra-day and inter-day precision) following guidelines from the US Food and Drug Administration (2015). For accurate quantification, a seven-point external standard calibration was employed, with each concentration analyzed in triplicate. Calibration curves were constructed by plotting the average peak area against the corresponding concentration (at least 7 concentrations). Sensitivity was established with limits of detection (LOD, S/N = 3) and quantification (LOQ, S/N = 10). Precision was evaluated at four concentration levels, with intra-day (*n* = 6 replicates) and inter-day (5 consecutive days) repeatability expressed as relative standard deviation (RSD). This rigorous validation ensured method reliability for quantitative phenolic profiling.

### 2.5 Determination of antioxidant activities and physicochemical parameters

The antioxidant activities of RO extracts were comprehensively evaluated using three validated assays: DPPH radical scavenging activity, ABTS radical cation decolorization, and ferric reducing antioxidant power (FRAP), performed according to the standardized protocols of Szydłowska-Czerniak and Łaszewska (2015). Colorimetric analysis was conducted using a precision colorimeter (WF32-16MM, WAVEGD, China) to determine the CIELAB color parameters (L* for lightness, a* for redness, and b* for yellowness). Oil yield was calculated gravimetrically as the percentage of extracted RO relative to the initial rapeseed mass. TPC of RO was determined using the Folin-Ciocalteu colorimetric method based on the protocol by Zhang et al. (2025) with minor modifications. Briefly, 50 μL of phenolic extract was mixed with 1.0 mL of Folin-Ciocalteu reagent in a 10 mL glass tube. After shaking for 3 min, 1.0 mL of saturated sodium carbonate solution and 7.95 mL of distilled water were added. The mixture was incubated in the dark at room temperature for 60 min, and absorbance was measured at 765 nm using a microplate reader (HBS-Scan X, DETIE, China). Quantification was performed against a sinapic acid calibration curve, with results expressed as mg sinapic acid equivalents per kg sample (mg/kg). Oil quality assessment included multiple oxidative stability indicators: acid value (AV, GB 5009.229–2016) for free fatty acid content (Gu et al., 2024), peroxide value (PV, GB 5009.227–2023) for primary oxidation products (Erol, Erdem, Yilmaz, Kayalar, & Cakli, 2022), and iodine value (IV, GB/T 5532–2022) for degree of unsaturation. Malondialdehyde (MDA) content, a sensitive marker of lipid peroxidation with potential cytotoxic effects, was determined spectrophotometrically (GB 5009.181–2016) to evaluate secondary oxidation products.

### 2.6 Analysis of fatty acid composition

Fatty acid profiling was conducted using a validated gas chromatographic method. Briefly, 2 mg of RO was precisely weighed into a 10 mL centrifuge tube and spiked with 70 μL of internal standard solution (5 mg/mL methyl nonadecanoate in n-hexane). Following the addition of 2 mL 0.4 mol/L KOH/methanol solution, the mixture was vortex-mixed for 10 min to ensure complete derivatization. The reaction was then quenched with 1 mL 0.9 % NaCl solution, and fatty acid methyl esters were extracted with 1 mL n-hexane through vigorous vortexing (1 min). After phase separation by centrifugation (5000 rpm, 5 min), the organic supernatant was collected for GC analysis. Fatty acid separation and quantification were performed using an Agilent 7820 A GC system (Agilent, USA) equipped with a flame ionization detector and a DB-23 column (30 m × 0.25 mm × 0.25 μm, Agilent, USA). Nitrogen served as the carrier gas. The column temperature program initiated at 50 °C, held for 2 min, then increased to 180 °C at a rate of 10 °C/min and held for 5 min. This was followed by a further increase to 230 °C at 5 °C/min, with a final hold for 5 min. The injection port temperature was set to 250 °C, with a pressure of 12.97 kPa, a split ratio of 15:1, a split flow rate of 12 mL/min, and a total flow rate of 14.4 mL/min. The detector temperature was maintained at 280 °C, with hydrogen and air flow rates of 30 mL/min and 300 mL/min, respectively, and a tail-blowing nitrogen flow rate of 25 mL/min. The injection volume was 2 μL. Fatty acids were identified by retention time matching against 37 authentic fatty acid methyl ester standards. Quantification employed internal standard calibration with methyl nonadecanoate. Data acquisition and processing were performed using LabSolutions software (Shimadzu, Japan).

### 2.7 Statistical analysis

All statistical analyses were conducted using SPSS 20.0 (IBM, USA) with statistical significance defined as *p* < 0.05. For comprehensive multivariate analysis, we employed MetaboAnalyst 5.0 (https://www.metaboanalyst.ca, Pang et al., 2021), conducting hierarchical clustering heatmap analysis, principal component analysis (PCA), partial least squares-discriminant analysis (PLS-DA), differential metabolite screening, and metabolic pathway analysis. Differential metabolites were identified based on combined criteria of statistical significance (*p* < 0.05) and variable importance in project (VIP ≥ 1). Correlation network diagram was obtained using Cytoscape 3.6.0. Data visualization was performed using Origin 8.6 (OriginLab, USA) and Microsoft Excel. All experimental procedures were conducted in triplicate, with results expressed as mean values ± standard deviation to ensure robust statistical interpretation.

## 3 Results and discussion

### 3.1 Development and validation of targeted metabolomics method

By investigating the fragmentation patterns of phenolic standards in tandem mass spectrometry, MRM transitions for 24 phenolic compounds were obtained. Authentic standards were used to optimize MS parameters, including precursor/product ions, Q1/Q3 pre-filters, and CEs. The MRM transitions and optimized MS parameters were detailed in **Table S2** (Supporting Information). For qualitative analysis, precursor ions, product ions, and retention times of phenolic compounds in RO were verified against authentic standards analyzed under identical LC-MS/MS conditions. Quantitative analysis was performed using the seven-point external standard calibration. Calibration results, including the regression equation, linear range, determination coefficient (R^2^), LODs, and LOQs for 24 phenolic compounds, were summarized in Table 1. All phenolic compounds exhibited excellent linearity, with R^2^ values exceeding 0.99. The method demonstrated exceptional sensitivity, with LODs (0.01–2.87 μg/kg) and LOQs (0.05–9.56 μg/kg) suitable for trace-level analysis. The method demonstrated high precision, with intra-day (0.64–15.99 % RSD) and inter-day (1.30–19.34 % RSD) precision well within acceptable limits (**Table S3**). These comprehensive validation results confirmed the method's reliability for accurate quantification of phenolic compounds in rapeseed oil matrices.

**Table 1:** Linear range, regression equation, R^2^, LODs, and LOQs of the method.

No.	Analyte	Linear range (μg/kg)	Regression equation	R^2^	LODs (μg/kg)	LOQs (μg/kg)
1	Sinapine	1–500	y = 85,193.9×-205,169	0.9997	0.1	0.32
2	Methyl sinapate	5–1000	y = 6128.5× + 12,066.1	0.9999	0.14	0.45
3	Coniferyl aldehyde	5–1000	y = 2642.7× + 5389.44	0.9999	0.22	0.73
4	Vanillin	5–1000	y = 333.512×-1130	0.9999	0.02	0.06
5	Baicalein	2–1000	y = 3759.74×-17,211.1	0.9999	0.02	0.05
6	Diosmetin	1–1000	y = 3582.27×-1030.98	0.9999	0.13	0.42
7	Naringenin	5–1000	y = 979.525×-4458.24	0.9998	0.24	0.81
8	Dihydroresveratrol	10–1000	y = 74.0659×-290.759	0.9985	1.59	5.3
9	Sinapic acid	10–100	y = 192.301× + 299.99	0.9999	2.5	8.35
10	Gallic acid	10–1000	y = 461.722× + 1128.82	0.9999	1.1	3.66
11	3,4-Dihydroxybenzoic acid	5–1000	y = 758.986× + 1973.12	0.9999	0.45	1.51
12	3-Hydroxycinnamic acid	5–1000	y = 992.076× + 3743.48	0.9999	0.89	2.98
13	4-Hydroxybenzoic acid	1–1000	y = 2924.45× + 437.939	0.9999	0.04	0.13
14	Apigenin	1–500	y = 2698.1× + 5133.18	0.9999	0.03	0.11
15	Astragalin	2–1000	y = 1320.33×-1678.22	0.9999	0.19	0.63
16	Genistein	1–200	y = 885.269× + 109.86	0.9994	0.14	0.47
17	Hesperetin	1–1000	y = 1139.35× + 6412.59	0.9993	0.13	0.43
18	Isorhamnetin	1–1000	y = 1427.01×-140.698	0.9999	0.12	0.4
19	Kaempferol	5–1000	y = 78.2164×-646.203	0.9997	2.87	9.56
20	Luteolin	1–1000	y = 1389× + 2791.48	0.9999	0.14	0.46
21	Morin	1–500	y = 3007.28× + 13,490.7	0.9994	0.03	0.09
22	Quercetin	1–500	y = 4421.47× + 17,013.4	0.9995	0.03	0.1
23	Hydroxytyrosol	2–1000	y = 672.095× + 6342.77	0.9997	0.15	0.51
24	Chlorogenic acid	1–1000	y = 1095.88× + 1204.7	0.9999	0.22	0.73

### 3.2 Phenolic metabolomic profile and chemometric analysis

**Fig. 1:**
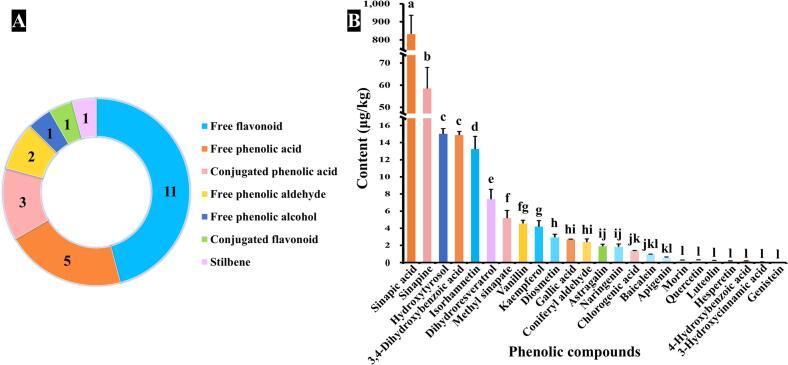
**A:** Types and numbers of phenolic compounds detected in RO. **B:** Contents of different phenolic compounds in RO. Results were expressed as mean values of three repetitions, and different letters in bar chart represent significant differences (*p* < 0.05).

Metabolomics provides a powerful tool for exploring phenolic profile changes in RO under varying processing and storage conditions. In this study, a total of 24 distinct phenolic compounds were identified and quantified, including 11 free flavonoids, 5 free phenolic acids, 3 conjugated phenolic acids, 2 free phenolic aldehydes, 1 free phenolic alcohol, 1 conjugated flavonoid, and 1 stilbene (Fig. 1A). Notably, our method detected 16 additional phenolic compounds compared to previous reports (Xu et al., 2020), demonstrating superior analytical sensitivity and coverage. The primary phenolic compounds identified in RO included sinapic acid (831.64 ± 104.26 μg/kg), sinapine (58.56 ± 9.45 μg/kg), hydroxytyrosol (15.02 ± 0.61 μg/kg), 3,4-dihydroxybenzoic acid (14.88 ± 0.42 μg/kg), and isorhamnetin (13.24 ± 1.50 μg/kg) (Fig. 1B). Among these, sinapic acid was the predominant free phenolic acid in RO, while sinapine, a product of the phenylalanine or hydroxycinnamate pathway, was the main esterified phenolic acid, contributing to the bitterness, astringency, and dark color of RO (Huang & Xu, 2020). And the measurement results were consistent with findings by Rękas, Ścibisz, Siger, and Wroniak (2017). Other phenolic compounds, such as gallic acid, baicalein, apigenin, and diosmetin, were also identified, compounds notable for their bioactive properties that contribute to health benefits (Nasiri et al., 2025; Sun, Li, & Zhang, 2025; Wang, Zhang, et al., 2021; Żurawek et al., 2025). The rich phenolic profile not only enhances the nutritional value of RO but also improves its oxidative stability, underscoring its importance as a functional food ingredient.

**Fig. 2:**
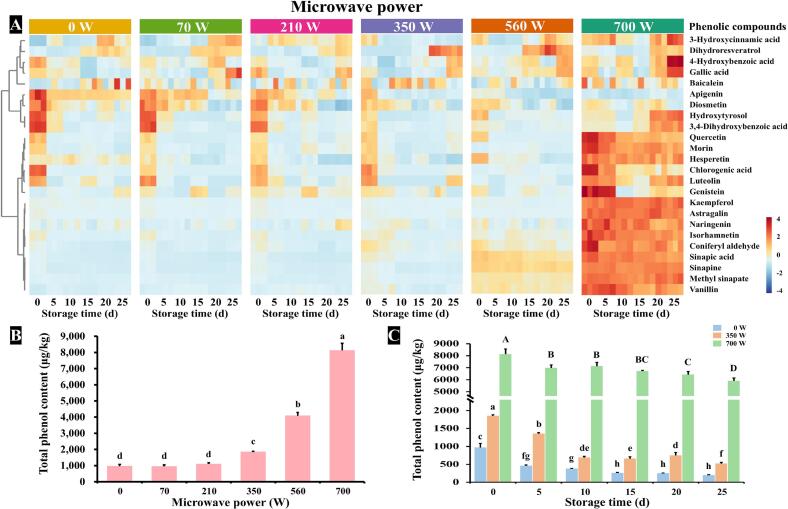
**A**: Hierarchical clustering heatmap analysis of phenolic compounds in RO under various microwave power and storage time. **B**: Effects of microwave power on the total phenol content of RO. **C**: Effects of storage time on the total phenol content of RO. Results in bar charts were expressed as mean values of three repetitions, and different letters on bars represent significant differences (*p* < 0.05).

To systematically elucidate the effects of microwave power and storage time on the phenolic profile of RO, we performed hierarchical clustering analysis and visualized the results as a heatmap (Fig. 2A). This analysis revealed three distinct clusters of phenolic compounds based on their response patterns. Cluster I, predominantly associated with 700 W microwave-treated samples, contained compounds including dihydroresveratrol, gallic acid, and baicalein. Cluster II, characterized by fresh oil samples, was enriched in apigenin, diosmetin, and hydroxytyrosol. Cluster III, comprising mainly 560–700 W treated samples, featured quercetin, sinapic acid, sinapine, and methyl sinapate. The heatmap analysis revealed that microwave power exerted a greater impact on phenolic profile of RO than storage duration. As shown in Fig. 2B, total phenol content of RO increased significantly with higher microwave power (*p* < 0.05), likely due to microwave-induced degradation of bound phenolic compounds and subsequent release of free forms into the oil matrix (Lee, Lee, Park, & Joo, 2025). Specific phenolic compounds, such as sinapine, methyl sinapate, coniferyl aldehyde, and quercetin, showed strong positive correlations with microwave power, while apigenin exhibited a negative correlation. In contrast, compounds such as baicalein, diosmetin, and luteolin showed no significant correlation with microwave power (*p* > 0.05), suggesting differential stability among phenolic compounds under microwave treatment.

Storage duration also influenced the phenolic compound levels of RO, with distinct degradation observed among different compounds. Notably, sinapine, sinapic acid, quercetin, and chlorogenic acid exhibited progressive declines during storage, likely due to their participation in antioxidant processes and oxidative degradation. This trend was aligned with previous research (Calatayud et al., 2024). In contrast, the concentration of dihydroresveratrol increased with extended storage time, possibly due to the metabolism of resveratrol (Jarosova et al., 2020). Interestingly, compounds such as vanillin and naringenin showed positive correlations with storage time at lower microwave power (70–350 W) but negative correlations at higher power (560–700 W). Moreover, phenolic compounds such as baicalein, gallic acid, and astragalin showed no significant correlation with either microwave power or storage duration. After 700 W microwave pretreatment, the total phenol content of RO increased by more than 7 times compared to untreated group. With the extension of storage time, the total phenol content of RO gradually decreased (Fig. 2C). After accelerated oxidation for 25 days, the total phenol content of 0, 350, and 700 W microwave pretreated RO decreased by 79.58 %, 71.96 %, and 27.46 %, respectively. It can be seen that the total phenol content in the 700 W microwave pretreated RO was the highest, and its loss of phenols was the smallest with the extension of storage time.

**Fig. 3:**
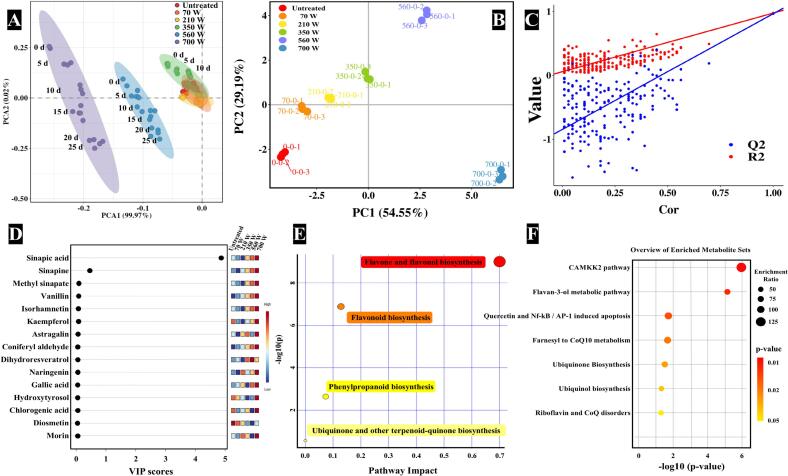
**A**: PCA score plot of phenolic compounds in RO under various microwave power and storage time. **B**: PLS-DA score plot of phenolic compounds in RO under various microwave powers. **C**: Permutation test plot. **D**: VIP score plot of phenolic compounds in RO under various microwave powers. **E**: Metabolic pathway analysis of phenolic compounds in RO samples. **F**: Pathway enrichment analysis of phenolic compounds in RO samples.

To comprehensively assess the differences and similarities in phenolic compounds among the RO samples, PCA was conducted (Cao, Zhang, Sun, Li, & Zheng, 2020). As illustrated in Fig. 3A, the PCA score plot demonstrated distinct clustering among RO samples treated at different microwave powers, with clear separation along two principal components. RO samples pretreated at lower microwave powers (210 W and 350 W) showed minimal separation from untreated samples, indicating similar phenolic profiles. However, as microwave power increased, the distribution of samples shifted leftward along the X-axis, allowing clear separation of samples pretreated at higher powers (560 W and 700 W). Additionally, prolonged storage duration under the same microwave power induced a gradual downward shift along the Y-axis (principal component 2), further facilitating sample differentiation. To enhance separation accuracy, PLS-DA was conducted (Fig. 3B), which effectively distinguished RO samples based on their phenolic profiles under different microwave powers. The first two principal components accounted for 54.55 % and 29.19 % of the total variance, respectively. A permutation test (Fig. 3C) confirmed the model's reliability, yielding a high Q2 value (0.966) with no indication of overfitting. Key differentiating compounds among RO samples were identified based on criteria of *p* < 0.05 and VIP ≥ 1 (Fig. 3D), with sinapic acid emerging as a key marker for distinguishing RO samples treated at different microwave powers. Sinapic acid, a primary metabolite in phenylpropanoid biosynthesis, plays a vital role in secondary metabolite production and is physiologically essential for growth, development, and reproduction. Beyond its biological significance, sinapic acid has practical applications as an antioxidant, fungicide, and anti-tumor agent. These findings underscored the substantial influence of microwave power and storage duration on the phenolic profile of RO, providing valuable insights for optimizing processing conditions to preserve or enhance bioactive compounds.

Metabolic pathway analysis was conducted using the KEGG database to explore the metabolic networks associated with phenolic compounds in RO (Wang et al., 2018). Several key pathways were identified, including flavone and flavonol biosynthesis, flavonoid biosynthesis, and phenylpropanoid biosynthesis, all of which exhibited significant associations (*p* < 0.01) with RO's phenolic composition (Fig. 3E). Pathway enrichment analysis (Fig. 3F) further highlighted highly enriched metabolic pathways, such as the CAMKK2 pathway, the quercetin and Nf-kB/AP-1 induced apoptosis pathway, and the farnesyl to CoQ10 metabolic pathway. These findings provided valuable insights into how microwave pretreatment and storage duration influence RO's phenolic profile by modulating specific metabolic pathways.

### 3.3 Antioxidant activities of phenolic extracts from RO

**Fig. 4:**
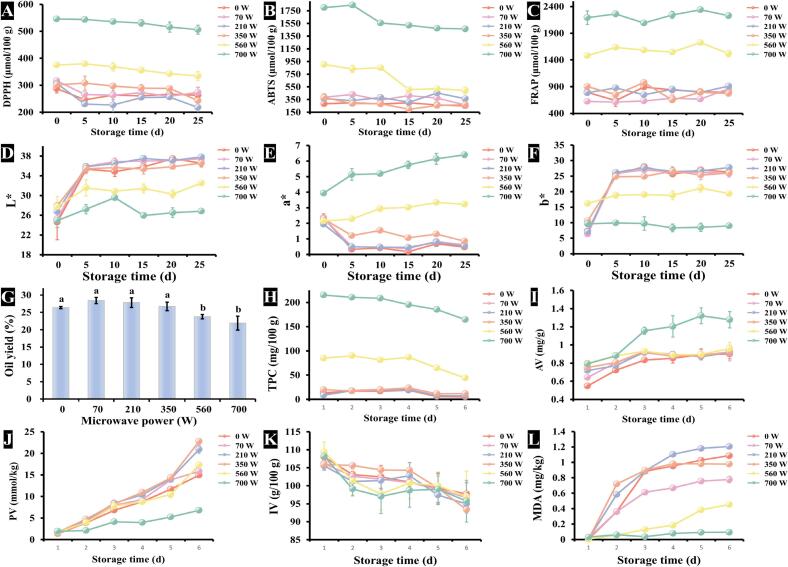
Changes in physicochemical characteristics of RO under various microwave power and storage time. **A**-**C** represent changes in the radical scavenging activities of DPPH**•**, ABTS^**·**+^, and FRAP, respectively. **D—F** represent changes in lightness (L*), redness (a*), and yellowness (b*) of RO, respectively. **G**: Effects of microwave power on the oil yield of RO. **H**: Effects of microwave power and storage time on TPC of RO. Results were expressed as mean values of three repetitions, and different letters in bar chart represent significant differences (*p* < 0.05). **I-L** represent changes in the AV, PV, IV, and MDA content of RO, respectively.

The antioxidant activities of phenolic extracts from RO subjected to different microwave pretreatments were evaluated throughout storage using DPPH radical scavenging (Fig. 4A), ABTS radical cation scavenging (Fig. 4B), and FRAP (Fig. 4C). The results demonstrated a clear positive correlation between microwave power and antioxidant capacity, with higher pretreatment powers (560 W and 700 W) yielding extracts with superior DPPH• and ABTS^·+^ scavenging capabilities as well as enhanced ferric ion reduction potential. Notably, phenolic extracts from samples pretreated at lower microwave powers (0 to 350 W), the antioxidant activities, including DPPH**•** and ABTS^**·**+^ scavenging capacities and FRAP, remained stable throughout the storage period. In contrast, the antioxidant activities of phenolic extracts from ROs pretreated at higher power (560 W and 700 W) exhibited distinct trends: DPPH**•** scavenging activity gradually declined, ABTS^**·**+^ scavenging activity decreased significantly, while FRAP remained relatively unchanged. These findings highlighted the influence of microwave pretreatment and storage duration on the antioxidant activities of RO phenolic extracts.

### 3.4 Physicochemical characteristics of ROs

Changes in the color of RO following different microwave pretreatments during storage were illustrated in Fig. 4D (L*), Fig. 4E (a*), and Fig. 4F (b*). Lightness and yellowness of RO pretreated at various microwave power levels exhibited similar trends over prolonged storage time. For oils pretreated at lower microwave powers (0 to 350 W), lightness and yellowness increased significantly during the first five days of storage and then stabilized from days 5 to 25. In contrast, RO samples pretreated at higher powers (560 W and 700 W) maintained relatively stable lightness and yellowness throughout the storage period. Redness, however, showed an inverse trend: it decreased significantly in ROs pretreated at lower microwave powers (0 to 350 W) within the first five days, followed by gradual stabilization from days 5 to 25. Conversely, the redness of ROs treated at 560 W and 700 W increased steadily over the storage period.

The relationship between microwave power and rapeseed oil yield was shown in Fig. 4G. The results indicated that oil yield initially increased with increasing microwave power, peaking at 70 W, before declining at higher power levels (70 to 700 W). This trend can be attributed to the fact that moderate microwave treatment can break down the cellular structures of rapeseed, enhancing oil release, while excessive power may damage the cells, reducing extraction efficiency. Notably, no significant changes in oil yield were observed between 0 and 350 W. However, from 350 W to 560 W, the oil yield decreased significantly, stabilizing again from 560 W to 700 W. These findings highlight the importance of optimizing microwave power to maximize both oil extraction efficiency and product quality.

Changes in TPC of RO following different microwave pretreatments during storage were presented in Fig. 4H. Results demonstrated a positive correlation between microwave power and TPC, with the highest phenolic content achieved at 700 W pretreatment. However, a gradual decline in TPC was observed throughout the storage period for all treatment groups.

Oxidative stability parameters of RO under various microwave pretreatment and storage conditions were evaluated through AV (Fig. 4I), PV (Fig. 4J), IV (Fig. 4K), and MDA content (Fig. 4L). AV, PV, and MDA levels increased with storage time, while IV decreased correspondingly. Notably, AV exhibited a rapid increase during the first 10 days before stabilizing, with the most pronounced increase observed in the 700 W treatment group. Importantly, all AV measurements remained below 1.4 mg/g, well within national quality standards. Similarly, the PV increased over time, but the rate of increase was slowest at 700 W, where the PV remained within the national quality standard range. This suggested that RO pretreated at 700 W exhibited the best oxidative stability. ROs pretreated at other microwave powers also met national standards during the first 10 days of storage. The IV of RO decreased gradually with extended storage, indicating a slight reduction in unsaturated fatty acid content, though the change was not significant. Whereas, MDA levels increased significantly during the first 10 days, followed by a slower increase over the next 15 days. Notably, treatments at 560 W and 700 W exhibited significantly slower MDA formation rates, further confirming their enhanced oxidative stability compared to lower power treatments.

The fatty acid composition of microwave-pretreated RO during storage was detailed in **Table S4**. The predominant fatty acids in RO, ranked in descending order of abundance: oleic acid (C18:1, 30.73 %–36.27 %) > erucic acid (C22:1, 21.43 %–26.30 %) > linolenic acid (C18:3, 10.73 %–23.00 %) > linoleic acid (C18:2, 11.00 %–14.51 %) > eicosenoic acid (C20:1, 7.25 %–9.01 %) > palmitic acid (C16:0, 2.60 %–3.50 %) > stearic acid (C18:0, 1.07 %–1.59 %) > arachidic acid (C20:0, 0.28 %–0.80 %). Notably, our findings differed from Huang et al. (2023), showing lower C18:1 but higher C18:3, C20:1, and C22:1 content, which may be related to differences in rapeseed varieties. The total fatty acid content remained stable across treatments, with untreated RO (1870.77–2668.53 mg/g) comparable to 700 W-pretreated RO (1926.62–2797.33 mg/g). As the microwave power increased, the content of C18:2 exhibited a significant decline, while the content of C20:0 significantly increased, which was similar to the findings reported by Özcan and Uslu (2023). It might be due to the thermal oxidation of unsaturated fatty acids and subsequent saturation, leading to a corresponding increase in saturated fatty acids. Other fatty acids showed no significant variations (*p* > 0.05), corroborating Wroniak et al. (2016). This comprehensive study demonstrated that microwave pretreatment and storage duration significantly affect RO's physicochemical properties, including color, oil yield, TPC, oxidative stability, and fatty acid composition. Each power level exhibited distinct effects, with 700 W emerging as the optimal pretreatment condition based on balanced performance metrics: maximizing phenolic content and oxidative stability while maintaining excellent oil yield and overall quality. These findings provided practical guidance for optimizing RO processing to enhance both nutritional value and shelf stability.

### 3.5 Correlation analysis of physicochemical properties and phenolic composition

**Fig. 5:**
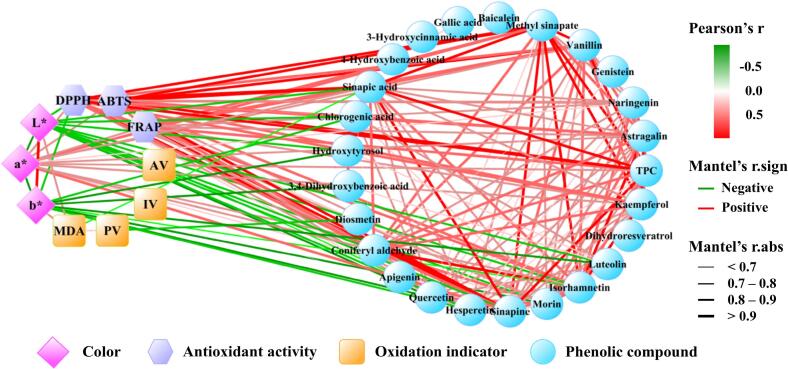
Correlation network diagram between phenolic compounds and physicochemical properties of RO. The red diamonds, purple hexagons, orange squares, and blue circles represent the color, antioxidant activities, oxidation indicators, and phenolic compounds of RO, respectively. Lines connected these variables, with red lines indicating positive correlations and green lines indicating negative correlations. The line thickness is proportional to the correlation strength, where thicker lines represent stronger associations. (For interpretation of the references to color in this figure legend, the reader is referred to the web version of this article.)

The correlation analysis (Fig. 5) demonstrated strong positive correlations (*p* < 0.01) among key phenolic compounds in RO, including sinapine, sinapic acid, methyl sinapate, coniferyl aldehyde, vanillin, isorhamnetin, astragalin, and kaempferol. Notably, color parameters showed distinct associations with phenolic content: lightness (L*) and yellowness (b*) of RO exhibited significant negative correlations (*r* < −0.7) with most phenolic compounds, such as quercetin, hesperetin, luteolin, morin, chlorogenic acid, and hydroxytyrosol. In contrast, redness (a*) showed strong positive correlations (*r* > 0.8) with TPC, antioxidant capacities (DPPH• and ABTS^·+^ scavenging capacities, and FRAP), and most phenolic compounds, including sinapine, methyl sinapate, sinapic acid, isorhamnetin, astragalin, kaempferol, vanillin, and coniferyl aldehyde. These findings suggested that higher phenolic content in RO was associated with deeper redness and enhanced antioxidant activity. Conversely, as oxidation progresses, phenolic compounds degrade, leading to reduced redness, increased lightness and yellowness, and diminished antioxidant capacity. Collectively, these findings underscored the complex interplay between RO's visual characteristics, phytochemical composition, and functional properties, providing valuable insights for quality assessment and processing optimization.

Correlation analysis revealed strong positive relationships (*r* > 0.9) between the antioxidant capacities of RO extracts, as measured by DPPH, ABTS, and FRAP assays, and multiple phenolic compounds, including sinapic acid, methyl sinapate, sinapine, vanillin, isorhamnetin, coniferyl aldehyde, kaempferol, astragalin, and naringenin. These results demonstrated the significant contribution of these phenolic compounds to RO's antioxidant potential, which aligned with numerous previous studies reporting similar positive associations between phenolic content and antioxidant activity (Eghlima et al., 2025; Moshari-Nasirkandi et al., 2024).

Regarding oxidation indicators, AV, PV, and MDA content showed negative correlations with specific phenolic compounds, while IV exhibited no significant relationships. Notably, higher apigenin levels correlated with lower AV (*r* < −0.6), while diosmetin and hesperetin were inversely associated with PV. MDA levels demonstrated negative correlations (r < −0.6) with both antioxidant capacities (DPPH• and ABTS^·+^ scavenging capacities) and multiple phenolic compounds, including sinapic acid, hesperetin, sinapine, quercetin, coniferyl aldehyde, methyl sinapate, isorhamnetin, and morin, suggesting their potential protective role against oxidative degradation. These comprehensive results underscored the intricate relationships between phenolic profile, antioxidant performance, color, and oxidation stability in RO, providing valuable insights for quality control and stability prediction in RO processing. However, the precise mechanisms and causal relationships underlying these observed associations warrant further investigation to fully elucidate their implications for RO stability and quality preservation.

## 4 Conclusions

In this study, a high accuracy targeted metabolomics method was developed and validated, and a total of 24 phenolic compounds were identified and quantified in RO. Chemometric analysis revealed that microwave power exerted a greater impact on phenolic profile of RO than storage duration. Specifically, higher microwave power and shorter storage times were associated with higher phenolic content. Total phenol content in the 700 W microwave pretreated RO was the highest, and its loss of phenols was the smallest with the extension of storage time. Sinapic acid was identified as a key marker for distinguishing RO samples treated at different microwave powers. Furthermore, flavone and flavonol biosynthesis, flavonoid biosynthesis, and phenylpropanoid biosynthesis pathways were significantly (*p* < 0.01) related to the phenolic compounds in RO. Changes in antioxidant capacity, color, TPC, oxidation degree, and fatty acid composition of RO following various microwave pretreatments and storage durations were also systematically examined. The results indicated that higher microwave power was associated with enhanced antioxidant capacity, redness, TPC, and AV, as well as decreased lightness, yellowness, PV, and MDA of RO samples. Longer storage duration was associated with higher AV, PV, and MDA, and lower IV and TPC. Correlation analysis revealed strong relationships between the phenolic profile and the antioxidant activity, color, and oxidation degree of RO. Specifically, the lightness and yellowness of RO exhibited significant negative correlations with most phenolic compounds, while redness showed strong positive correlations with TPC, antioxidant capacities, and most phenolic compounds. Overall, these findings enhanced our understanding of the intricate relationships between phenolic composition, antioxidant activity, color, and oxidative stability of RO, providing valuable insights into factors influencing the quality and stability of RO. Future research will systematically investigate the direct relationships between phenolic compound profiles and the key quality parameters in RO, including antioxidant capacity, nutritional value, and oxidative stability. Building on the current findings, we will specifically examine: (1) the structure-activity relationships of phenolic compounds as natural antioxidants in edible oils; (2) their potential toxicity thresholds and bioavailability under various processing conditions; and (3) their physiological mechanisms of action in human metabolism. This comprehensive research will provide critical scientific evidence to evaluate both the health benefits and safety considerations of phenolic-fortified edible oils for nutritional applications, ultimately supporting the development of value-added functional food products with optimized phenolic compositions.

## Statement of consent

Before carrying out experiments, participants were informed about the background, process, possible benefits and risks of this research. Written informed consent was obtained from all the participants prior to the enrollment of this study.

## CRediT authorship contribution statement

**Yao Zhang:** Writing – original draft, Methodology, Investigation, Formal analysis. **Zhixian Xiang:** Methodology, Investigation. **Rong Xia:** Methodology, Investigation. **Wenxi Chen:** Visualization, Investigation. **Xin Zhang:** Investigation, Data curation. **Hongting Lei:** Investigation. **Fang Wei:** Writing – review & editing, Validation. **Yongjun Yuan:** Supervision, Investigation.

## Declaration of competing interest

The authors declare that they have no known competing financial interests or personal relationships that could have appeared to influence the work reported in this paper. All authors have seen the manuscript and approved to submit to your journal.

## Data Availability

No data was used for the research described in the article.

## Appendix A Supplementary data

Supplementary material Image 1
