# Supplementary material for: Comprehensive analysis of phenolic profile, their antioxidant activities, and physicochemical characteristics of rapeseed oil under various microwave and storage conditions
Source: Food Chem X. 2025 Jun 11;29:102637. doi: 10.1016/j.fochx.2025.102637 (PMC12205591; doi:10.1016/j.fochx.2025.102637)
Supplement: Supplementary file 1 — Supplementary material [file mmc1.docx]

*Supporting information for*

**Comprehensive analysis of phenolic profile, their antioxidant activities, and physicochemical characteristics of rapeseed oil under various microwave and storage conditions**

Yao Zhang^a,b 1^, Zhixian Xiang^a,b 1^, Rong Xia^a,b^, Wenxi Chen^a,b^, Xin Zhang^a,b^, Hongting Lei^a,b^, Fang Wei^c,d*^, Yongjun Yuan^a,b*^

^a^Chongqing Key Laboratory of Speciality Food Co-Built by Sichuan and Chongqing, School of Food and Bioengineering, Xihua University, Chengdu 610039, China

^b^Key Laboratory of Grain and Oil Processing and Food Safety of Sichuan Province, College of Food and Bioengineering, Xihua University, Chengdu 610039, China

^c^Oil Crops Research Institute of Chinese Academy of Agricultural Sciences, Key Laboratory of Oilseeds Processing of Ministry of Agriculture, Hubei Key Laboratory of Lipid Chemistry and Nutrition, Wuhan 430062, China

^d^Hubei Hongshan Laboratory, Wuhan 430070, China

Corresponding author:

*(Fang Wei) E-mail: willasa@163.com.

Address: Oil Crops Research Institute of Chinese Academy of Agricultural Sciences, Wuhan, 430062, China.

*(Yongjun Yuan) E-mail: yyja9791@sina.com.

Address: School of Food and Bioengineering, Xihua University, Chengdu, 610039, China.

Tel.: 028-87720037

Fax: 028-87720552

The supporting information includes the following items:

**Figure S1.** Temperature of rapeseed after different microwave times.

**Table S1.** Information of phenolic standards.

**Table S2.** MRM transitions and MS parameters of the method.

**Table S3.** Intra-day and inter-day precision of the method.

Table S4. Fatty acid composition of rapeseed oil after microwave pretreatment during storage.

# Figure S1


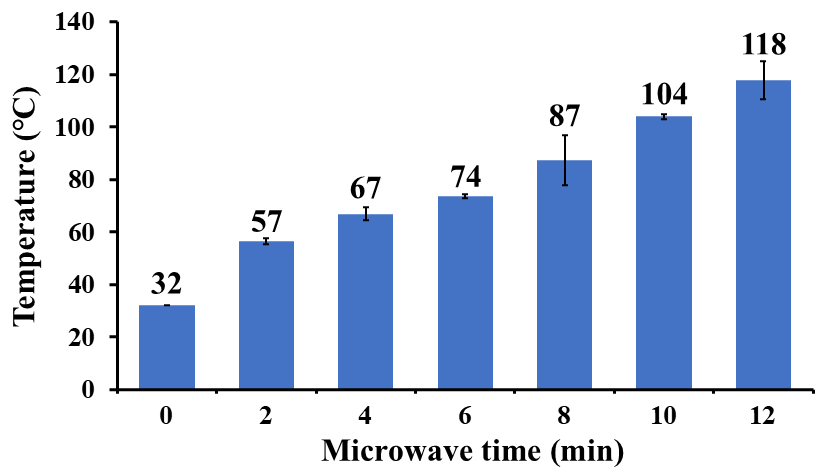


# Table S1. Information of phenolic standards.

| No. | Name | Formula | Molecular Weight | CAS | Purity | Producer |
| --- | --- | --- | --- | --- | --- | --- |
| 1 | [Sinapine](https://www.chemsrc.com/en/cas/18696-26-9_406282.html) | C_16_H_24_NO_5_ | 310.3655 | 18696-26-9 | 99.90% | ANPEL Laboratory Technologies (Shanghai) Inc. |
| 2 | Methyl sinapate | C_12_H_14_O_5_ | 238.2407 | 20733-94-2 | 98.90% | ANPEL Laboratory Technologies (Shanghai) Inc. |
| 3 | Coniferyl aldehyde | C_10_H_10_O_3_ | 178.1851 | 20649-42-7 | 98.50% | ANPEL Laboratory Technologies (Shanghai) Inc. |
| 4 | Vanillin | C_8_H_8_O_3_ | 152.1470 | 121-33-5 | 99.90% | ANPEL Laboratory Technologies (Shanghai) Inc. |
| 5 | Baicalein | C_15_H_10_O_5_ | 270.2370 | 491-67-8 | 98.00% | ANPEL Laboratory Technologies (Shanghai) Inc. |
| 6 | Diosmetin | C_16_H_12_O_6_ | 300.2631 | 520-34-3 | 98.10% | ANPEL Laboratory Technologies (Shanghai) Inc. |
| 7 | Naringenin | C_15_H_12_O_5_ | 272.2530 | 67604-48-2 | 99.70% | ANPEL Laboratory Technologies (Shanghai) Inc. |
| 8 | Dihydroresveratrol | C_14_H_14_O_3_ | 230.2593 | 58436-28-5 | 99.00% | ANPEL Laboratory Technologies (Shanghai) Inc. |
| 9 | Sinapic acid | C_11_H_12_O_5_ | 224.2100 | 530-59-6 | 98.00% | ANPEL Laboratory Technologies (Shanghai) Inc. |
| 10 | Gallic acid | C_7_H_6_O_5_ | 170.1195 | 149-91-7 | 98.80% | ANPEL Laboratory Technologies (Shanghai) Inc. |
| 11 | 3,4-Dihydroxybenzoic acid | C_7_H_6_O_4_ | 154.1201 | 99-50-3 | 98.40% | ANPEL Laboratory Technologies (Shanghai) Inc. |
| 12 | 3-Hydroxycinnamic acid | C_9_H_8_O_3_ | 164.1580 | 588-30-7 | 99.90% | ANPEL Laboratory Technologies (Shanghai) Inc. |
| 13 | 4-Hydroxybenzoic acid | C_7_H_6_O_3_ | 138.1210 | 99-96-7 | 99.90% | ANPEL Laboratory Technologies (Shanghai) Inc. |
| 14 | [Apigenin](https://www.chemsrc.com/en/cas/520-36-5_254337.html) | C_15_H_10_O_5_ | 270.2370 | 520-36-5 | 99.00% | ANPEL Laboratory Technologies (Shanghai) Inc. |
| 15 | Astragalin | C_21_H_20_O_11_ | 448.3770 | 480-10-4 | 99.90% | ANPEL Laboratory Technologies (Shanghai) Inc. |
| 16 | [Genistein](https://www.chemsrc.com/en/cas/446-72-0_57659.html) | C_15_H_10_O_5_ | 270.2370 | 446-72-0 | 99.20% | ANPEL Laboratory Technologies (Shanghai) Inc. |
| 17 | Hesperetin | C_16_H_14_O_6_ | 302.2790 | 520-33-2 | 99.90% | ANPEL Laboratory Technologies (Shanghai) Inc. |
| 18 | [Isorhamnetin](https://www.chemsrc.com/en/cas/480-19-3_749632.html) | C_16_H_12_O_7_ | 316.2650 | 480-19-3 | 99.40% | ANPEL Laboratory Technologies (Shanghai) Inc. |
| 19 | [Kaempferol](https://www.chemsrc.com/en/cas/520-18-3_894792.html) | C_15_H_10_O_6_ | 286.2360 | 520-18-3 | 98.90% | ANPEL Laboratory Technologies (Shanghai) Inc. |
| 20 | [Luteolin](https://www.chemsrc.com/en/cas/491-70-3_950941.html) | C_15_H_10_O_6_ | 286.2390 | 491-70-3 | 99.20% | ANPEL Laboratory Technologies (Shanghai) Inc. |
| 21 | Morin | C_15_H_10_O_7_ | 302.2360 | 480-16-0 | 98.50% | ANPEL Laboratory Technologies (Shanghai) Inc. |
| 22 | Quercetin | C_15_H_10_O_7_ | 302.2360 | 117-39-5 | 97.60% | ANPEL Laboratory Technologies (Shanghai) Inc. |
| 23 | Hydroxytyrosol | C_8_H_10_O_3_ | 154.1630 | 10597-60-1 | 98.70% | ANPEL Laboratory Technologies (Shanghai) Inc. |
| 24 | Chlorogenic acid | C_16_H_18_O_9_ | 354.3090 | 327-97-9 | 99.50% | ANPEL Laboratory Technologies (Shanghai) Inc. |

# Table S2. MRM transitions and MS parameters of the method.

| No. | Analyte | Retention time | Scan mode | Precursor ion (*m/z*) | Product ion (*m/z*) | Q1 Pre (V) | Q3 Pre (V) | CE (V) |
| --- | --- | --- | --- | --- | --- | --- | --- | --- |
| 1 | Sinapine | 1.193 | [M+H] ^+^ | 311.20 | 252.20 | -22 | -25 | -16 |
| 2 | Methyl sinapate | 3.141 | [M+H] ^+^ | 239.15 | 207.15 | -17 | -21 | -9 |
| 3 | Coniferyl aldehyde | 2.474 | [M+H] ^+^ | 179.10 | 91.00 | -13 | -29 | -24 |
| 4 | Vanillin | 2.100 | [M+H] ^+^ | 153.05 | 65.05 | -11 | -24 | -26 |
| 5 | Baicalein | 3.371 | [M+H] ^+^ | 271.05 | 122.95 | -19 | -21 | -35 |
| 6 | Diosmetin | 3.281 | [M+H] ^+^ | 301.15 | 286.05 | -11 | -30 | -27 |
| 7 | Naringenin | 3.154 | [M+H] ^+^ | 272.95 | 153.00 | -10 | -28 | -21 |
| 8 | Dihydroresveratrol | 2.571 | [M+H] ^+^ | 231.05 | 137.25 | -16 | -14 | -15 |
| 9 | Sinapic acid | 1.938 | [M-H] ^−^ | 223.00 | 208.05 | 11 | 21 | 12 |
| 10 | Gallic acid | 1.188 | [M-H] ^−^ | 169.00 | 125.15 | 18 | 12 | 15 |
| 11 | 3,4-Dihydroxybenzoic acid | 1.198 | [M-H] ^−^ | 153.10 | 108.95 | 18 | 19 | 15 |
| 12 | 3-Hydroxycinnamic acid | 2.347 | [M-H] ^−^ | 162.95 | 119.05 | 18 | 20 | 13 |
| 13 | 4-Hydroxybenzoic acid | 2.609 | [M-H] ^−^ | 137.30 | 93.05 | 15 | 17 | 16 |
| 14 | Apigenin | 3.177 | [M-H] ^−^ | 268.95 | 117.00 | 30 | 19 | 35 |
| 15 | Astragalin | 1.753 | [M-H] ^−^ | 447.15 | 284.05 | 23 | 18 | 28 |
| 16 | Genistein | 3.207 | [M-H] ^−^ | 268.95 | 133.15 | 13 | 25 | 30 |
| 17 | Hesperetin | 3.274 | [M-H] ^−^ | 301.15 | 163.95 | 15 | 29 | 25 |
| 18 | Isorhamnetin | 3.318 | [M-H] ^−^ | 315.25 | 300.05 | 16 | 30 | 20 |
| 19 | Kaempferol | 3.222 | [M-H] ^−^ | 285.15 | 186.85 | 30 | 20 | 30 |
| 20 | Luteolin | 2.752 | [M-H] ^−^ | 285.15 | 133.00 | 21 | 23 | 36 |
| 21 | Morin | 2.777 | [M-H] ^−^ | 301.25 | 151.05 | 15 | 28 | 22 |
| 22 | Quercetin | 2.776 | [M-H] ^−^ | 300.95 | 151.05 | 15 | 29 | 21 |
| 23 | Hydroxytyrosol | 1.197 | [M-H] ^−^ | 153.15 | 109.00 | 16 | 10 | 13 |
| 24 | Chlorogenic acid | 1.171 | [M-H] ^−^ | 353.10 | 191.10 | 18 | 20 | 18 |

# Table S3. Intra-day and inter-day precision of the method.

| No. | Analyte | Intra-day precision (RSD%, n=6) | | | | Inter-day precision (RSD%, n=5) | | | |
| --- | --- | --- | --- | --- | --- | --- | --- | --- | --- |
|  |  | 10 μg/kg | 50 μg/kg | 100 μg/kg | 200 μg/kg | 10 μg/kg | 50  μg/kg | 100 μg/kg | 200 μg/kg |
| 1 | Sinapine | 4.15 | 2.58 | 2.52 | 3.44 | 5.05 | 2.07 | 8.83 | 3.93 |
| 2 | Methyl sinapate | 6.19 | 3.93 | 4.70 | 5.73 | 14.71 | 8.38 | 7.78 | 4.90 |
| 3 | Coniferyl aldehyde | 1.45 | 8.34 | 2.45 | 3.02 | 4.68 | 10.25 | 8.23 | 1.85 |
| 4 | Vanillin | 3.79 | 1.35 | 15.99 | 5.62 | 3.46 | 7.05 | 9.23 | 13.96 |
| 5 | Baicalein | 4.37 | 1.47 | 7.47 | 10.37 | 12.12 | 14.71 | 10.27 | 13.46 |
| 6 | Diosmetin | 8.13 | 10.03 | 7.65 | 4.85 | 8.04 | 9.87 | 10.59 | 6.62 |
| 7 | Naringenin | 4.31 | 4.17 | 9.18 | 4.43 | 12.11 | 12.74 | 4.79 | 7.05 |
| 8 | Dihydroresveratrol | 15.47 | 12.16 | 9.67 | 0.73 | 16.58 | 8.76 | 9.27 | 15.07 |
| 9 | Sinapic acid | 10.04 | 8.44 | 5.79 | 3.44 | 9.54 | 11.89 | 12.14 | 17.38 |
| 10 | Gallic acid | 11.79 | 4.80 | 5.05 | 11.90 | 12.57 | 14.60 | 9.27 | 8.20 |
| 11 | 3,4-Dihydroxybenzoic acid | 8.77 | 2.41 | 0.64 | 4.79 | 13.56 | 9.54 | 6.33 | 12.87 |
| 12 | 3-Hydroxycinnamic acid | 6.12 | 8.33 | 6.46 | 12.71 | 4.07 | 9.36 | 11.35 | 10.09 |
| 13 | 4-Hydroxybenzoic acid | 8.24 | 12.78 | 9.17 | 5.25 | 10.86 | 12.07 | 15.52 | 16.59 |
| 14 | Apigenin | 3.89 | 11.15 | 2.85 | 4.88 | 1.30 | 13.96 | 5.38 | 4.12 |
| 15 | Astragalin | 2.80 | 13.61 | 5.34 | 6.63 | 16.21 | 5.35 | 6.47 | 3.50 |
| 16 | Genistein | 6.13 | 2.22 | 9.14 | 6.78 | 9.08 | 16.50 | 6.54 | 6.82 |
| 17 | Hesperetin | 3.42 | 5.08 | 5.98 | 5.90 | 16.21 | 8.75 | 6.81 | 8.92 |
| 18 | Isorhamnetin | 5.18 | 3.21 | 10.21 | 14.98 | 8.50 | 7.67 | 15.94 | 4.09 |
| 19 | Kaempferol | 6.72 | 3.31 | 4.06 | 4.04 | 10.84 | 9.33 | 15.52 | 13.03 |
| 20 | Luteolin | 3.95 | 5.18 | 12.37 | 7.79 | 16.44 | 12.24 | 11.87 | 5.22 |
| 21 | Morin | 3.79 | 11.54 | 7.05 | 6.44 | 12.88 | 12.63 | 5.60 | 7.35 |
| 22 | Quercetin | 1.22 | 9.74 | 6.59 | 5.71 | 8.59 | 11.19 | 7.06 | 6.74 |
| 23 | Hydroxytyrosol | 10.77 | 3.51 | 2.03 | 13.16 | 11.07 | 14.40 | 16.55 | 6.46 |
| 24 | Chlorogenic acid | 5.80 | 3.39 | 9.56 | 9.18 | 13.56 | 9.72 | 4.50 | 8.32 |

# Table S4. Fatty acid composition of rapeseed oil after microwave pretreatment during storage.

| Microwave power  and storage time | Content (%) | | | | | | | |
| --- | --- | --- | --- | --- | --- | --- | --- | --- |
|  | C16:0 | C18:0 | C18:1 | C18:2 | C18:3 | C20:0 | C20:1 | C22:1 |
| 0 W, 0 d | 3.27±0.05ab | 1.36±0.05abcdefgh | 34.89±0.71abcde | 13.06±0.26abcd | 15.25±0.75hijkl | 0.55±0.06bc | 7.95±0.43bcdef | 23.66±0.16efghij |
| 0 W, 5 d | 3.29±0.11a | 1.41±0.02abcde | 34.52±0.30abcdefg | 12.95±0.01abcdef | 16.14±1.07fghi | 0.40±0.05efghij | 8.00±0.20bcdef | 23.28±0.70ghijkl |
| 0 W, 10 d | 3.21±0.14abcd | 1.26±0.06efghij | 35.60±0.56ab | 12.99±0.25abcde | 13.83±0.47klmn | 0.36±0.04fghij | 7.85±0.40def | 24.89±0.52bc |
| 0 W, 15 d | 3.15±0.03abcdef | 1.33±0.03bcdefgh | 35.21±0.06abc | 12.91±0.01abcdefg | 13.55±0.15lmn | 0.42±0.01efgh | 8.51±0.04ab | 24.91±0.04bc |
| 0 W, 20 d | 3.26±0.11ab | 1.39±0.11abcdefg | 35.23±0.19abc | 13.20±0.44ab | 12.03±1.22n | 0.34±0.03hij | 8.63±0.32a | 25.91±0.37a |
| 0 W, 25 d | 3.13±0.11abcdefg | 1.20±0.12hij | 33.81±1.06cdefghij | 13.15±1.18abc | 14.24±0.92ijklm | 0.45±0.02def | 8.21±0.19abcde | 25.80±0.47ab |
| 70 W, 0 d | 3.25±0.13ab | 1.30±0.08cdefghij | 34.31±0.94bcdefgh | 12.30±0.51defghijk | 16.64±1.36efgh | 0.53±0.09bcd | 8.19±0.07abcde | 23.47±0.84efghijkl |
| 70 W, 5 d | 3.14±0.13abcdef | 1.48±0.01ab | 32.95±0.56hij | 12.68±0.27abcdefghi | 17.86±0.98cdefg | 0.41±0.04efghi | 8.16±0.22abcdef | 23.31±0.59ghijkl |
| 70 W, 10 d | 3.18±0.13abcde | 1.33±0.08bcdefgh | 34.98±0.43abcd | 13.30±0.24a | 13.94±1.27jklmn | 0.38±0.04efghij | 8.32±0.40abcd | 24.57±0.97cde |
| 70 W, 15 d | 3.01±0.08defghi | 1.24±0.12fghij | 33.32±1.45fghij | 12.21±0.54efghijk | 19.40±1.75bc | 0.39±0.06efghij | 8.00±0.33bcdef | 22.43±0.88l |
| 70 W, 20 d | 3.06±0.07bcdefg | 1.28±0.11defghij | 33.13±0.77ghij | 12.16±0.26fghijkl | 19.01±1.58bcd | 0.34±0.05hij | 7.91±0.17cdef | 23.12±0.47hijkl |
| 70 W, 25 d | 3.12±0.07abcdefg | 1.33±0.19bcdefgh | 34.00±0.49cdefghi | 12.41±0.27bcdefghijk | 17.51±0.34cdefgh | 0.41±0.05efghi | 8.01±0.03bcdef | 23.20±0.79ghijkl |
| 210 W, 0 d | 3.19±0.11abcd | 1.36±0.02abcdefgh | 34.24±0.39bcdefgh | 12.74±0.15abcdefghi | 17.06±1.04defgh | 0.60±0.01b | 8.04±0.12bcdef | 22.77±0.34ijkl |
| 210 W, 5 d | 3.31±0.18a | 1.52±0.06a | 34.33±0.82bcdefgh | 12.67±0.35abcdefghi | 16.95±0.95defgh | 0.55±0.04bc | 7.85±0.02def | 22.83±0.38ijkl |
| 210 W, 10 d | 3.20±0.13abcd | 1.32±0.06bcdefgh | 35.58±0.86ab | 12.59±0.45abcdefghij | 15.82±0.83fghijk | 0.43±0.02efgh | 8.01±0.25bcdef | 23.05±0.74hijkl |
| 210 W, 15 d | 3.03±0.05cdefgh | 1.16±0.09ij | 33.69±0.76defghij | 12.21±0.31efghijk | 18.09±1.77cdef | 0.47±0.02cde | 7.98±0.21bcdef | 23.37±0.56ghijkl |
| 210 W, 20 d | 3.19±0.03abcd | 1.39±0.08abcdefg | 35.81±0.43a | 12.92±0.17abcdefg | 13.03±0.81mn | 0.40±0.04efghij | 8.44±0.12abc | 24.81±0.10cd |
| 210 W, 25 d | 3.07±0.12bcdefg | 1.41±0.06abcdef | 33.47±1.72efghij | 12.36±0.34cdefghijk | 16.44±1.93fgh | 0.43±0.03efgh | 8.36±0.15abcd | 24.46±0.07cdef |

Note: values were showed as means ± standard deviations (n = 3), different letters in the same column represent significant differences (*p* < 0.05).

**Table S4.** Continued

| Microwave power  and storage time | Content (%) | | | | | | | |
| --- | --- | --- | --- | --- | --- | --- | --- | --- |
|  | C16:0 | C18:0 | C18:1 | C18:2 | C18:3 | C20:0 | C20:1 | C22:1 |
| 350 W, 0 d | 3.13±0.04abcdef | 1.35±0.02bcdefgh | 33.99±0.38cdefghi | 12.64±0.14abcdefghij | 17.73±0.36cdefg | 0.60±0.02b | 8.03±0.10bcdef | 22.53±0.36kl |
| 350 W, 5 d | 3.31±0.06a | 1.46±0.04abc | 33.91±0.69cdefghij | 12.75±0.38abcdefgh | 16.84±1.98defgh | 0.41±0.06efghi | 7.89±0.36cdef | 23.42±0.50efghijkl |
| 350 W, 10 d | 3.15±0.16abcdef | 1.34±0.04bcdefgh | 34.82±0.60abcde | 12.44±0.60bcdefghij | 16.32±1.32fghi | 0.35±0.05ghij | 7.92±0.32cdef | 23.67±0.29efghij |
| 350 W, 15 d | 2.95±0.04fghi | 1.26±0.02efghij | 32.61±0.43ij | 11.86±0.16jkl | 20.55±0.66ab | 0.44±0.06efg | 7.73±0.12ef | 22.60±0.13jkl |
| 350 W, 20 d | 3.11±0.04abcdefg | 1.31±0.06cdefghi | 34.59±0.59abcdef | 12.72±0.37abcdefghi | 15.98±0.77fghij | 0.32±0.02ij | 8.27±0.26abcde | 23.70±0.54efghi |
| 350 W, 25 d | 3.06±0.04bcdefg | 1.44±0.07abcd | 32.60±1.33ij | 12.00±0.48hijkl | 17.97±1.81cdefg | 0.56±0.08b | 7.83±0.42def | 24.54±0.54cde |
| 560 W, 0 d | 3.22±0.03abcd | 1.39±0.00abcdefg | 34.66±0.31abcdef | 12.75±0.11abcdefgh | 15.79±0.42ghijk | 0.59±0.01b | 8.22±0.04abcde | 23.37±0.16ghijkl |
| 560 W, 5 d | 3.17±0.22abcdef | 1.44±0.05abcd | 34.40±0.83bcdefg | 13.06±0.42abcd | 16.14±0.92fghi | 0.35±0.04ghij | 8.11±0.09abcdef | 23.34±0.35ghijkl |
| 560 W, 10 d | 3.13±0.10abcdefg | 1.33±0.05bcdefgh | 33.90±0.26cdefghij | 12.39±0.11cdefghijk | 17.42±0.40cdefgh | 0.40±0.06efghij | 8.01±0.05bcdef | 23.44±0.30efghijkl |
| 560 W, 15 d | 3.17±0.02abcde | 1.24±0.07fghij | 33.90±0.26cdefghij | 12.28±0.15defghijk | 17.00±0.07defgh | 0.47±0.06cde | 8.00±0.12bcdef | 23.94±0.59cdefgh |
| 560 W, 20 d | 3.10±0.05abcdefg | 1.34±0.14bcdefgh | 33.50±0.33efghij | 12.10±0.21hijkl | 17.67±1.15cdefg | 0.31±0.01j | 7.89±0.16cdef | 24.09±0.74cdefgh |
| 560 W, 25 d | 2.97±0.08efghi | 1.38±0.12abcdefg | 32.58±0.35ij | 11.65±0.16kl | 19.39±1.14bc | 0.43±0.06efgh | 7.84±0.26def | 23.78±0.93defghi |
| 700 W, 0 d | 3.20±0.19abcd | 1.33±0.09bcdefgh | 33.95±0.52cdefghi | 12.14±0.98ghijkl | 16.61±0.98efgh | 0.68±0.10a | 8.16±0.48abcdef | 23.93±0.51cdefgh |
| 700 W, 5 d | 3.24±0.15abc | 1.40±0.11abcdefg | 33.79±0.47cdefghij | 12.23±0.46efghijk | 17.20±1.66cdefgh | 0.41±0.05efghij | 8.15±0.80abcdef | 23.58±0.69efghijk |
| 700 W, 10 d | 2.82±0.20i | 1.24±0.05ghij | 34.07±0.87cdefgh | 12.08±0.18hijkl | 17.19±0.82cdefgh | 0.42±0.03efgh | 7.90±0.57cdef | 24.28±0.24cdefg |
| 700 W, 15 d | 2.85±0.03hi | 1.14±0.04j | 31.30±0.54k | 11.41±0.19l | 21.99±1.44a | 0.40±0.05efghij | 7.61±0.14f | 23.31±0.90ghijkl |
| 700 W, 20 d | 2.92±0.05ghi | 1.30±0.18cdefghij | 32.50±0.98j | 11.94±0.43ijkl | 18.78±1.13bcde | 0.43±0.06efgh | 8.02±0.34bcdef | 24.12±0.40cdefgh |
| 700 W, 25 d | 3.11±0.07abcdefg | 1.38±0.06abcdefg | 33.11±0.19ghij | 11.95±0.15hijkl | 17.78±0.40cdefg | 0.58±0.06b | 8.06±0.05bcdef | 24.02±0.09cdefgh |

Note: values were showed as means ± standard deviations (n = 3), different letters in the same column represent significant differences (*p* < 0.05).
